# Physiologically-Based Pharmacokinetic/Pharmacodynamic Model of MBQ-167 to Predict Tumor Growth Inhibition in Mice

**DOI:** 10.3390/pharmaceutics12100975

**Published:** 2020-10-15

**Authors:** Javier Reig-López, María del Mar Maldonado, Matilde Merino-Sanjuan, Ailed M. Cruz-Collazo, Jean F. Ruiz-Calderón, Victor Mangas-Sanjuán, Suranganie Dharmawardhane, Jorge Duconge

**Affiliations:** 1 Department of Pharmacy and Pharmaceutical Technology and Parasitology, Faculty of Pharmacy, University of Valencia, 46100 Burjassot, Valencia, Spain; jareiglo@alumni.uv.es (J.R.-L.); matilde.merino@uv.es (M.M.-S.); 2 Department of Biochemistry, School of Medicine, University of Puerto Rico, Medical Sciences Campus, San Juan, PR 00936, USA; mariadelmar.maldonado@upr.edu (M.d.M.M.); ailed.cruzcollazo@upr.edu (A.M.C.-C.); jean.ruiz7@upr.edu (J.F.R.-C.); su.d@upr.edu (S.D.); 3 Interuniversity Research Institute for Molecular Recognition and Technological Development, 46100 Burjassot, Valencia, Spain; 4 School of Pharmacy, University of Puerto Rico, Medical Sciences Campus, San Juan, PR 00936, USA; jorge.duconge@upr.edu

**Keywords:** breast cancer, MBQ-167, physiologically based pharmacokinetic modeling, Rac inhibitor

## Abstract

MBQ-167 is a dual inhibitor of the Rho GTPases Rac and Cdc42 that has shown promising results as an anti-cancer therapeutic at the preclinical stage. This drug has been tested in vitro and in vivo in metastatic breast cancer mouse models. The aim of this study is to develop a physiologically based pharmacokinetic/pharmacodynamic (PBPK-PD) model of MBQ-167 to predict tumor growth inhibition following intraperitoneal (IP) administration in mice bearing Triple Negative and HER2+ mammary tumors. PBPK and Simeoni tumor growth inhibition (TGI) models were developed using the Simcyp V19 Animal Simulator. Our developed PBPK framework adequately describes the time course of MBQ-167 in each of the mouse tissues (e.g., lungs, heart, liver, kidneys, spleen, plasma) and tumor, since the predicted results were consistent with the experimental data. The developed PBPK-PD model successfully predicts tumor shrinkage in HER2+ and triple-negative breast tumors after the intraperitoneal administration of 1 and 10 mg/kg body weight (BW) dose level of MBQ-167 three times a week. The findings from this study suggest that MBQ-167 has a higher net effect and potency inhibiting Triple Negative mammary tumor growth compared to HER2+ and that liver metabolism is the major route of elimination of this drug.

## 1. Introduction

Drug discovery and development represents an increasing economic and temporal cost for the pharmaceutical industry, which has not translated into significant increases in the number of approved active ingredients, especially in the oncology area [1,2]. One alternative is to develop mathematical models at the preclinical stages of the drug development process capable of better predicting efficacy or safety outcomes in order to efficiently design clinical trials [3]. Physiologically- based pharmacokinetic (PBPK) modelling represents a mathematical framework that integrates physicochemical, physiological, and biochemical information to predict the concentration-time course at target tissues for a wide range of exposure conditions in animals or humans [4]. In recent years, the use of PBPK models has clearly improved the model-informed drug discovery and development process of several drugs [5,6,7], which has facilitated its recognition by the main regulatory agencies (FDA and EMA) [8,9]. Currently, the main purposes of PBPK models are to qualitatively and quantitatively predict drug-drug interactions and to support initial dose selection in pediatric and first-in-human trials [8].

The tumor growth inhibition (TGI) model [10] constitutes a highly valuable preclinical methodology in oncology for the selection of therapeutic candidates and the design of optimal clinical evaluation strategies for the in vivo evaluation of anti-tumor effect [11,12,13,14,15,16]. The Simeoni TGI model has been widely implemented to characterize the pharmacological response of drug candidates in single-agent and combination experiments by linking drug concentration in the target tissue to the inhibition of tumor growth [17].

The small molecule MBQ-167 is an anticancer therapeutic candidate that inhibits breast cancer metastasis in vivo and has been characterized as a potent inhibitor of the Rho GTPases Rac and Cdc42 [18]. These GTPases are overactive in different cancer types [19,20,21,22,23] and promote cancer cell migration, invasion, proliferation, and oncogenic transformation. MBQ-167 dually inhibits the activation of both GTPases, with half-maximal inhibitory concentrations (*IC*_50_) of 0.1 μM and 0.08 μM for Rac and Cdc42, respectively. Preclinical studies have shown that MBQ-167 inhibits breast cancer cell migration, viability, tumor growth, and metastasis in vivo without apparent toxicity [18,24]. Currently, this compound is being developed for clinical applications as a potential anti-metastatic therapeutic. Nonetheless, further studies are needed to characterize the tumoral pharmacokinetics (PK) of MBQ-167, which is essential to improve therapy efficacy and success rate further [25].

Therefore, the aims of this work were: (i) to develop a PBPK model of MBQ-167 after intraperitoneal (IP) administration in mice, and (ii) to characterize tumor growth dynamics in two human breast cancer cell lines (HER2+ and Triple Negative).

## 2. Materials and Methods

### 2.1. Materials

MBQ-167 and EHop-0036 (internal standard) were synthesized as previously described [18,26]. Purity (>98%) was verified by thin-layer chromatography (TLC), nuclear magnetic resonance (NMR) and gas chromatography/mass spectrometry. Sodium chloride, ethyl acetate, heptane, acetonitrile, methanol, and all materials required for compound synthesis were also purchased from Sigma-Aldrich (St. Louis, MO, USA). Formic acid was purchased from Fisher (Fair Lawn, NJ, USA).

### 2.2. Animal Protocol

All animal studies were conducted under approved animal protocols (#A8180117, #A8180112) by the University of Puerto Rico Medical Sciences Campus Institutional Animal Care and Use Committee, in accordance with the principles and procedures outlined in the NIH Guideline for the Care and Use of Laboratory Animals [27]. Four to five-week-old female BALB/c mice (Charles River Laboratories, Inc. Wilmington, MA, USA) were housed under pathogen-free conditions in HEPA-filtered cages and kept on a 12 h light/dark cycle, and controlled temperature (22–24 °C), and humidity (25%). Food and water were given ad libitum. MBQ-167 was prepared as a stock solution of 2 mg/mL in Cremophor^®^:ethanol:PBS (12.5:12.5:75) solution. Each mouse was administered a single 0.1 mL dose of MBQ-167 (in 12.5% ethanol, 12.5% Cremophor^®^, 75% phosphate buffered saline, pH 7.4) that corresponded to 10 mg/kg body weight (BW) by IP injection.

### 2.3. Tumor Pharmacokinetics

Thirty-five BALB/c mice were injected with 2.5 × 10^5^ 4T1 murine metastatic breast cancer cells at the mammary fat pad under isoflurane inhalation (1–3% in oxygen using an inhalation chamber at 2 L/min) to produce primary tumors, as described [28]. Following IP treatment with a single dose of MBQ-167 (10 mg/kg), five mice/group were sacrificed by cervical dislocation at 0.5, 1, 3, 6, 9,12, and 24 h. Following sacrifice, tumors were collected and flushed with normal saline, individually wrapped in aluminum foil, snap-frozen in liquid nitrogen, and stored frozen at −80 °C until use.

### 2.4. Tumor Sample Preparation

Tumor samples were extracted by liquid-liquid extraction method, as previously described by [26]. Briefly, frozen tumors were thawed, weighed (100 mg), and homogenized using the Polytron PT 2100 instrument in pH 7.4 saline (1:4 *w/v*). Tumor homogenate (100 μL) was then transferred to another tube and the internal standard EHop-0036 (10 μL from 4500 ng/mL stock) was added to the samples followed by vortex (30 s). A hundred microliters (100 μL) of sodium hydroxide 0.5 M were then added to the mixture and samples were mixed by vortex for 5 min. Afterwards, 790 μL of heptane: ethyl acetate mixture (1:1) were added and samples were vortexed again for 10 min. The upper layer was recovered following centrifugation (5 min at 510 × *g*) and the solvent was evaporated for one hour in a Centrivap console (Labconco, Kansas City, MO, USA) at room temperature. Samples were then reconstituted with 100 μL of methanol, vortexed for ten minutes, and centrifuged at 1000× *g* for 1 min.

### 2.5. Instrumentation

We used a validated bioanalytical method using supercritical fluid chromatography coupled with tandem mass spectrometry to quantify MBQ-167 in tumors and tissues, as previously described [26]. The analysis was performed on an Acquity UPC^2^ system (Waters Corp., Milford, MA, USA) coupled to a triple quadrupole tandem mass spectrometer (MS/MS). An Acquity UPC^2^ BEH (3.0 × 100 mm^2^, 1.7 μm) column was used for separation purposes.

### 2.6. Her2+/Triple Negative Tumor Growth Study

As published elsewhere [18], female athymic nude (nu/nu) mice, 4 to 5 weeks old (Charles River Laboratories, Inc., Wilmington, MA, USA) were maintained under pathogen-free conditions in HEPA-filtered cages (5 mice per cage) under controlled light (12 h light and dark cycle), temperature (22–24 °C), and humidity (25%).

Mammary fat pad tumors were established using green fluorescent protein (GFP)-tagged MDA-MB-435 (HER2++) cells in Matrigel (BD Biosciences, San Jose, CA, USA) or GFP-MDA-MB-231 (TNBC) cells by injecting at the fourth right mammary fat pad under isofluorane inhalation (1–3% in oxygen using an inhalation chamber at 2 L/min) to produce orthotopic primary tumors. After tumor establishment (1-week post-inoculation), the animals from the same litter with similar weight and tumor size were randomly divided into experimental treatment groups (*n* = 10 per treatment group).

Mice were treated with vehicle (12.5% ethanol, 12.5% Cremophor^®^ (Sigma-Aldrich, St. Louis, MO, USA), and 75% 1X PBS pH 7.4), or 1, 5, or 10 mg/kg BW MBQ-167 by IP injection in a 100 µL volume every other day, three times a week. Treatments continued until sacrifice at day 65 for the HER2+ tumors and day 108 for the Triple Negative cell line.

### 2.7. Whole Body Fluorescence Image Analysis

Mammary tumor growth was quantified as changes in the integrated density of green fluorescent protein (GFP) fluorescence. Mice were imaged one week following breast cancer cell inoculation (on day 1 of treatment administration) and once a week thereafter. The FluorVivo small animal in vivo imaging system (INDEC Systems, Inc., Santa Clara, CA, USA) was used for whole body imaging of GFP fluorescence. Tumor fluorescence intensities were analyzed using Image J software (National Institutes of Health, Bethesda, MD, USA, 2019, Version 1.52q 13). Relative tumor growth was calculated as the integrated density of fluorescence of each tumor on each day of imaging relative to the integrated density of fluorescence of the same tumor on day 1 of treatment administration, relative to vehicle controls.

### 2.8. Physiologically Based Pharmacokinetic Model

#### 2.8.1. Modelling Strategy

**Figure 1:**
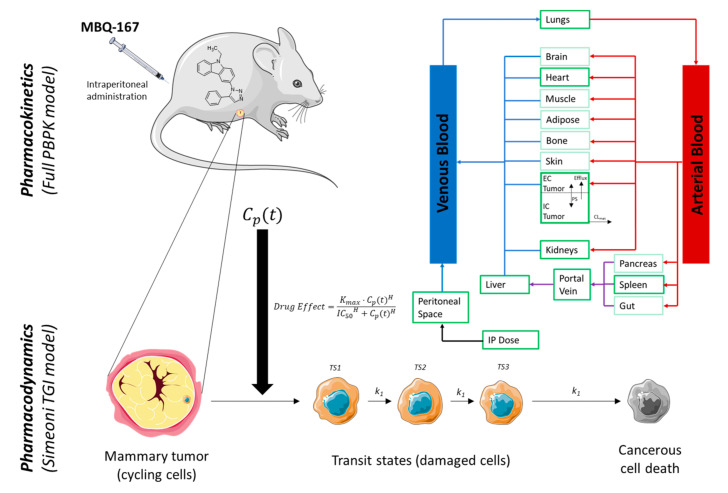
MBQ-167 PBPK-PD model structure. Light green compartments were included in the full PBPK distribution model but not evaluated in the present work. EC: extracellular water. IC: intracellular water. Efflux: clearance of the efflux transporter. *PS*: passive permeability clearance between intra- and extracellular water. *CL*_met_: metabolic clearance at the tumor tissue. *C*p(t): plasma concentration over time. *K*_max_: maximum inhibition. *IC*_50_: concentration associated with *K*_max_/2. H: Hill coefficient. TS1: transit State 1. TS2: transit State 2. TS3: transit State 3. *k*_1_: transit rate cell damage constant. This figure was created using Servier Medical Art templates (https://smart.servier.com/) which are licensed under a Creative Commons Attribution 3.0 Unported License [29].

The PBPK model (Figure 1) of MBQ-167 in mice after IP administration was developed in Simcyp Animal Simulator (Certara UK Limited, Sheffield, UK, 2020, V19). Physicochemical properties and both in vitro and in vivo ADME data were incorporated into the software for evaluating the drug’s exposure and response dynamics. The modelling strategy (“middle-out” approach) is briefly described as follows and fully depicted in Supplementary Material Figure S1, Pharmacokinetics.

Parameter estimation (PE) was performed using the PE Module of the Simcyp Animal V19 using the Nelder-Mead method, weighted least squares by the reciprocal of square of maximum observed value as the objective function and the termination criteria defined as the improvement of less than 1% of the objective function value. Optimization was manually performed to best fit the observed data.

The physiological parameters of the typical mouse were modified to reproduce the mice population used in the experimental procedure. Initial parameter estimation of the fraction unbound in plasma (*fu*) and blood-to-plasma (*B/P*) ratio was performed based on the reported systemic plasma clearance and volume of distribution of MBQ-167 [26], assuming an intravenous bolus injection of 30 s, since no IP route of administration is explicitly implemented in Simcyp V19 Animal Simulator. 

After defining the kinetics of the IP absorption process, systemic plasma clearance was predicted by scaling the intrinsic clearance from in vitro hepatocytes. Optimization of the tissue-to-plasma partition coefficients (*Kp_t_*) led to improve predictions of the base PBPK model and extrapolate them to other tissues such as the lungs, liver, kidneys, heart, and spleen (advanced PBPK model). Finally, modeling of the tumor disposition provided the final MBQ-167 PBPK model. The key input parameters for the PBPK model of MBQ-167 are summarized in Table 1.

#### 2.8.2. Physicochemical Properties and Plasma Binding 

The molecular weight of MBQ-167 is 338.414 g/mol and the water partition coefficient (log*P*_octanol:water_) ratio is 4.944. MBQ-167 is a monoprotic base compound with a p*K*_a_ of 0.27. The *f_u_* in plasma was estimated to 0.02. *B/P* ratio was initially estimated in the base PBPK model development phase and then optimized using a local sensitivity analysis (LSA) with a final value of 1.8.

#### 2.8.3. Absorption

Since Simcyp V19 Animal Simulator lacks of an explicitly implemented IP route of administration, the IP absorption was described through a first-order process, which included an absorption rate constant (*k_a_*) that regulated the absorption to the venous blood, assuming an IP bioavailability of 100%, avoiding gut and liver first-pass effect and pre-systemic degradation. In this sense, *k_a_* value was optimized to 3 h^−1^ to characterize maximum plasma concentration (*C*_max_) and time to maximum plasma concentration (*T*_max_). Lag time (0.17 h) was incorporated to best reproduce the absorption process.

#### 2.8.4. Distribution

Rodger and Rowland’s method (method 2) within a full PBPK model distribution was used. *Kp_t_* parameters of the heart, kidney, liver, lungs, and spleen were optimized based on the observed data from each tissue. Final values are provided in Table 1. A *Kp_t_* scalar of 0.29 predicted a volume of distribution at steady state (*V_ss_*) of 20.21 L/Kg, which is in good agreement with that reported in the literature of 400 mL [26] for a mice population of 20 g of body weight.

#### 2.8.5. Elimination

Due to the limited information of any active process in MBQ-167 renal excretion and/or tubular reabsorption, it was assumed that renal excretion was only mediated by glomerular filtration. For this reason, renal clearance (*CL_R_*) was set as the mean basal glomerular filtration rate for mice (12–18 mL/min) [26]. The metabolism of MBQ-167 in the liver includes up to nine metabolites when incubated in liver microsomes and that inhibits CYP3A4, 2C9, 2C19 and 1A2, but it is unknown through which isoenzyme this metabolism occurs. In this sense, liver metabolism was estimated based on the intrinsic clearance from in vitro hepatocytes (79 mL/min/10^6^ cells) and optimizing the incubation unbound fraction of the drug (*f_u_inc_*) to best predict the reported systemic plasma clearance value (2.15 mL/min).

#### 2.8.6. Tumor Disposition

Initial parameter estimation of the passive permeability clearance between intra- and extracellular water (*PS*) and the intrinsic clearance of an efflux transporter was performed followed by an optimization of these values to best fit the observed data. Final values were set as 1.2 mL/min/mL tumor volume and 7 mL/min/mL tumor volume for PS and intrinsic clearance of the efflux transporter, respectively. MBQ-167 tumor intrinsic clearance (*CL*_tumor_) was implemented to improve exposure predictions with a value of 2.2 mL/min/mL tumor volume. Vascularization was set as 16% of the tumor volume.

#### 2.8.7. Population

Physiological parameters of the typical mouse were modified to reproduce the mice population used in the experimental procedure. In this sense, body weight, tissue volumes, blood flows, cardiac output, and liver and tumor density were adapted to the studied population.

#### 2.8.8. Simulation Trials

Typical mouse predictions were generated and individual simulations in the fed state were performed after a single MBQ-167 IP dose of 10 mg/kg of body weight for 12 h (plasma and tissues) and 24 h (tumor) duration.

### 2.9. MBQ-167 PBPK Model Verification

The final MBQ-167 PBPK model was verified through both graphical and numerical analysis. Experimental and predicted longitudinal plasma concentration- (*C_p_*) and tissue concentration- (*C*_t_) profiles were generated, including the 95% confidence intervals (95% CI) of the observations at each sampling time and the mean predicted concentrations. LSA was performed to evaluate the relative impact of *B/P*, *f_u_*, *CL*_R_, *CL*_tumor_ and *PS* in the plasma PK exposure parameters (*AUC*_0-t_ and *C*_max_). The performance of the MBQ-167 PBPK model was assessed by the fold error at each tissue, which referred to the ratio of the predicted *AUC*_0-t_ or *C*_max_ to the observed *AUC*_0-t_ or *C*_max_, respectively (Equation (1)). *AUC*_0-t_ was calculated by the trapezoidal rule. Both graphical and numerical analyses were performed in RStudio version 1.2.5019 (Boston, MA, USA, 2019) with R version 3.5.1 (GNU project).

(1) $$Fold\;ERROR\;PK\;parameter=\frac{Predicted\;PK\;parameter}{Observed\;PK\;parameter}$$

### 2.10. MBQ-167 Tumor Growth Inhibition Model Development

An unperturbed and perturbed Simeoni tumor growth models [10] were developed within the Simcyp V19 Animal Simulator for two breast cancer (BC) cell lines, e.g., HER2+ and Triple Negative (Supplementary Material Figure S1, Pharmacodynamics). First, the parameters governing tumor growth, e.g., exponential growth rate (λ_0_), linear growth rate (λ_1_) and shape factor (Ψ), were estimated from in vivo experiments of tumor growth volume from the control group of HER2+ and Triple Negative cell lines, respectively (unperturbed model). Once characterized tumor growth, the inhibition model was developed. Linear and non-linear response models, as well as total plasma and whole tumor concentration as the input for the drug effect were tested. A parameter estimation including all dose levels (1 and 10 mg/Kg for the HER2+ and for the Triple Negative cell lines) was performed to estimate the parameters governing this perturbed model: maximum inhibition (*K*_max_), concentration associated with half *K*_max_/2 (*IC*_50_) and transit rate of cell damage (*k*_1_). The number of transit compartments was established regarding the promptness appearance of the tumor growth inhibition for each cell line. Initial tumor volume was theoretically set as 0.1 mL for the HER2+ cell line. In the case of the Triple Negative cell line, initial tumor volume was estimated using MBQ-167 and vehicle treated mice. Final tumor growth inhibition (TGI) model parameters are shown in Table 2.

Model evaluation of tumor kinetics was performed by calculating the relative error (RE) (Equation (2)):

(2) $$RE\left(\%\right)=\frac{PRED\;Volume-\bar{OBS\;Volume}}{\bar{OBS\;Volume}}\cdot 100\;\;\;\;\;\;\;\;\;\;\;\;\;\;$$

## 3. Results

### 3.1. MBQ-167 PBPK Model

**Figure 2:**
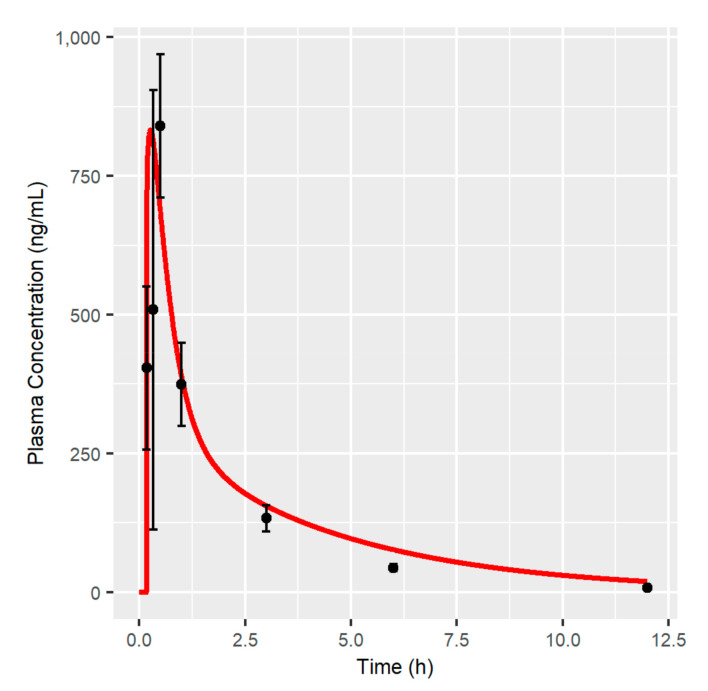
Plasma concentration-time profile after the IP single dose administration of 10 mg/kg of MBQ-167 in mice. Red line represents the typical simulated individual. Black dots represent the mean of all observations at the sample time with the corresponding 95% Confidence Interval (vertical lines).

**Figure 3:**
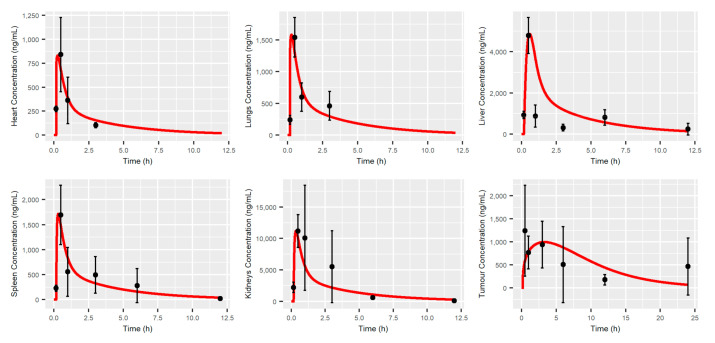
Tissue and tumor concentration-time profiles after the IP single dose administration of 10 mg/kg of MBQ-167 in mice. Red line represents the typical simulated individual. Black dots represent the mean of all observations at the sample time with the corresponding 95% Confidence Interval (vertical lines).

Model predictions after a single IP administration of 10 mg/kg of MBQ-167 are shown in Figure 2; Figure 3, showing that the PBPK model developed is able to capture the longitudinal MBQ-167 observations. *C*_max_ and *T*_max_ were adequately characterized in plasma and other organs (heart, lungs, liver, spleen, and kidneys), and slightly under-estimated the *C*_max_ and over-estimated the *T*_max_ in tumor tissue. These results agree with the numerical analysis (Table 3), as the fold error for *C*_max_ is close to the unity in all tissues except from tumor, where a value of 0.8 arises. *AUC*_0-t_ fold errors were within the 20% accepted range (0.8–1.2) for heart (1.02), lungs (0.97), spleen (0.81), and tumor (1.12). Liver *AUC*_0-t_ was slightly over-predicted, with an *AUC*_0-t_ fold error of 1.36, possibly due to the over-prediction of exposure between 1 and 5 h after the administration of MBQ-167 (Figure 3). In order to balance the deviation caused by the PBPK model in the liver, the predictions in the kidney are under-estimated, leading to an *AUC*_0-t_ fold error in kidneys equal to 0.63. However, it must be noted that the simulated typical profile matched the 95% CI of the observations in all sample times (Figure 3).

The PBPK model developed predicts a systemic plasma clearance of 2.13 mL/min with a clear dominance of liver metabolism (1.83 mL/min) over renal excretion (0.3 mL/min) of MBQ-167.

### 3.2. MBQ-167 Tumor Growth Inhibition Model

**Figure 4:**
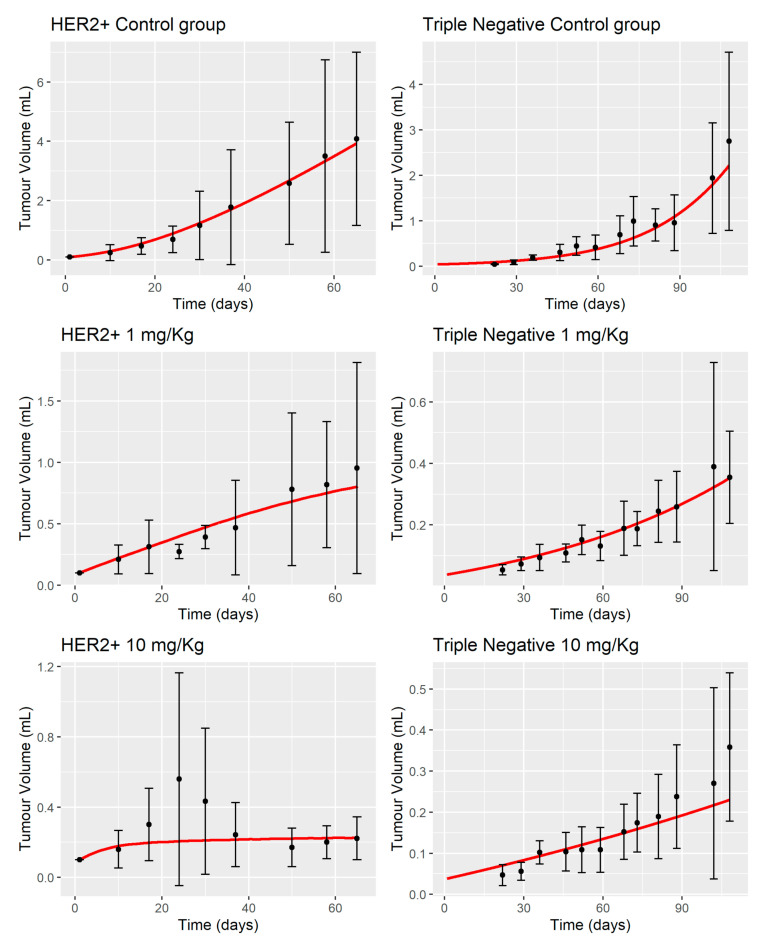
Tumor growth model (perturbed and unperturbed) for the HER2+ and Triple Negative cell lines. Red line represents the typical simulated individual. Black dots represent the mean of all observations at the sample time with the corresponding 95% Confidence Interval (vertical lines).

**Figure 5:**
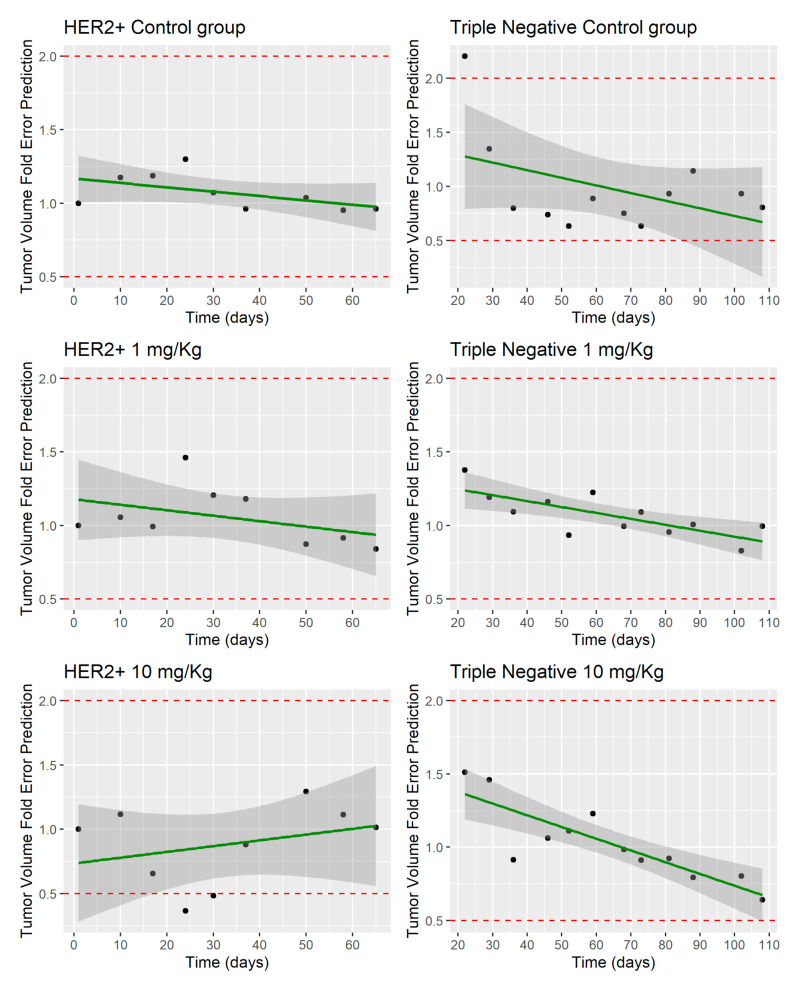
Evaluation of tumor volume predictions in unperturbed and perturbed (1 and 10 mg/kg) groups in HER2+ and Triple Negative cell lines. Red dashed lines represent the validation range (0.5 and 2-fold error). Green solid line represents the linear regression of the data. Black dots represent the fold-error with the mean profile for each group.

Figure 4 shows the PBPK-PD predictions of tumor growth for the HER2+ and Triple Negative cell lines in the absence of MBQ-167 (control group) and treated groups at 1 and 10 mg/kg BW dose levels. The unperturbed tumor growth models proposed by Simeoni et al. [10] (Figure 4) are capable of describing tumor growth with no antitumoral activity in both cell lines (control groups). In addition, Figure 4 depicts the predicted tumor growth dynamics in HER2+ and Triple Negative cell lines after the administration of MBQ-167 (perturbed model) at 1 and 10 mg/kg BW dose levels, respectively. The TGI models are in good agreement with observed data, with the predicted tumor growth profile within the 95% CI of all observations of the study. Figure 5 represents the fold-error difference between the mean observed profile and model predictions. Based on the RE, the structural PBPK-PD is able to predict tumor kinetics of the control groups and the 1 mg/kg groups of HER2+ and Triple Negative cell lines, indicating that the mean tendency is adequately captured by the model. However, an under-prediction was observed in the tumor profile of HER2+ cell line at 10 mg/kg between days 10 and 30.

The parameters governing the exponential growth (λ_0_) were 0.2 and 0.039 day^−1^, which shows a more sustained and prolonged growth of the Triple Negative cell line in this phase. However, the zero-order process (λ_1_) of Triple Negative was higher (0.5457 g/day) compared to HER2+ cells (0.12 g/day). Different initial tumor volumes were assumed for the HER2+ cell line (0.1 mL) compared to Triple Negative (0.0384 mL) in order to properly capture the exponential growth in both cell lines. A different number of damaged cell compartments were considered in order to address the delay of death with respect to the drug treatment, assuming progressive damage of tumor cells. The different number of damaged compartments for each cell line has characterized, in a flexible manner, the different death rates of tumor cells for each cell line, allowing to a more sustained- and prolonged tumor growth inhibition in Triple Negative cell line (*n* = 4).

Different drug effect parameters (*k*_1_ and *K*_max_/*IC*_50_) were considered for each cell line, showing a higher potency of MBQ-167 in Triple Negative cells (*IC*_50_ = 0.0001 μM) compared to HER2+ cell line (*IC*_50_ = 0.0187 μM). The net effect, which results from the ration between *K*_max_/*IC*_50_, was higher for the Triple Negative cell line (533 mL/ng·day^−1^) compared to the HER2+ cell line (19.7 mL/ng·day^−1^).

## 4. Discussion

A PBPK-PD model in mice for the recently developed Rac/Cdc42 inhibitor MBQ-167 has been developed that is able to describe both the pharmacokinetic and pharmacodynamic properties of the drug. The PBPK-PD model properly describes the time course of MBQ-167 in plasma and other tissues (e.g., lungs, heart, liver, kidneys, spleen) and predicts tumor growth inhibition when administered to mice in two BC cell lines, HER2+ and Triple Negative. The model implements the IP administration of MBQ-167, full-body distribution, hepatic metabolism, and renal excretion. Furthermore, the model considers a permeability-limited tumor distribution and implements the Simeoni TGI model to assess the antitumoral effect.

### 4.1. MBQ-167 PBPK Model

The developed PBPK framework is capable of adequately describing the time course of MBQ-167 in each of the mouse tissues. The relative error in *AUC*_0-t_ and *C*_max_ in each of the tissues is, in general, less than 20% for the typical profile, which shows that the PBPK model is capable of characterizing the average trend of behavior.

The time to reach maximum concentration (*T*_max_) through IP route resulted in 0.26 h, showing a rapid absorption that is similar to other reported anticancer small molecules [30,31,32]. The model was able to fit all observations prior to 6 h, with a little overprediction from this time up to 12 h. Probably, the overprediction in the terminal phase of the *C*_p_-time profile justifies the slight difference between predicted (2.13 mL/min) and observed (2.15 mL/min) systemic plasma clearance. The rapid elimination of MBQ-167 from plasma, with a predicted elimination half-life (*t*_1/2_) of 2.98 h after IP administration of 10 mg/kg BW, is consistent with other reports of Rac inhibitors like EHop-016 or EHT1864, with *t*_1/2_ values of 5.73 h [30] and 1.65 h [33], respectively. Predicted plasma exposition PK parameters *AUC*_0-t_ and *C*_max_ were remarkably close to that observed, with fold errors of 1.09 and 0.99, respectively, showing the optimal prediction performance of the model. The predicted *Vss* (20.21 L/kg) reveals high distribution into peripheral tissues, with little remaining of MBQ-167 in the bloodstream [26].

Tissue distribution of MBQ-167 was assessed plotting the corresponding concentration-time profiles in the liver, lungs, heart, kidneys, and spleen, verifying their fitting to the 95% CI of the observed values at each sample time and through the computing of the fold error for *AUC*_0-t_ and *C*_max_. The highest concentration of MBQ-167 was found in the kidneys, with a *C*_max_ value of 11231.7 ng/mL, reflecting the important role of renal uptake and subsequent clearance in drug disposition [26]. The predicted rank order of tissue drug exposure, determined by both *AUC*_0-t_ and *C*_max_ was kidneys > liver > spleen > lung > heart. Tumor concentration was adequately predicted by the model, with *AUC*_0-t_ and *C*_max_ fold errors in the desired range, and the predicted typical profile correctly fitting the observed data. However, a slight difference in the tumor disposition process is evident, with a predicted rate of distribution slower than that observed, as is determined by lower and longer *C*_max_ and *T*_max_, respectively.

Regarding the elimination mechanisms, the PBPK-PD model predictions reveal liver metabolism as the major route of elimination, as it represents 86% of overall systemic clearance. However, these results must be handled with care as no additional information is known about renal excretion and only glomerular filtration of unaltered MBQ-167 has been implemented in the model.

### 4.2. MBQ-167 Tumor Growth Inhibition Model

Tumor growth dynamics of two cell lines of breast cancer (HER2+ and Triple Negative) were modelled in the absence (unperturbed) or presence (perturbed) of MBQ-167 using the model proposed by Simeoni et al. [10]. There is vast scientific evidence regarding the ability of the model to quantitatively characterize the time-course of tumor dynamics in xenograft experiments and evaluate the efficacy of anticancer drugs early in discovery and development [15,34,35,36,37,38,39,40]. The mathematical framework allowed for an adequate prediction of the tumor dynamics under the different groups considered.

The PBPK-PD model is able to successfully predict tumor volume (*RE* < 20%) in the unperturbed group of both cell lines. The TGI model accurately predicts tumor shrinkage (*RE* < 20%) in HER2+ breast cancer cell line after the administration of 1 and 10 mg/kg BW of MBQ-167 three times a week for 65 days in mice, with a relative tumor size reduction of 94.3% at the highest dose level. Model predictions in Triple Negative cell lines agreed with the experimental data for the 1 mg/kg group, and a slightly under-prediction of final tumor volume was predicted in mice receiving 10 mg/kg three times a week for 108 days. The discrepancy could be explained by the fact that differences in λ_1_ could appear between groups, but the overall time-course profile of tumor dynamics of each group is adequately captured by the model since mean predictions are within the 95% CI of the observed data and the predicted relative reduction in the final tumor size (89.6%) agrees with that observed (87.0%) at the 10 mg/kg BW dose level. It has been demonstrated that MBQ-167 reduces mammary fat pad tumor size starting approximately 3 weeks following treatment at a nontoxic concentration of 10 mg/kg BW, and resulting in total inhibition of metastases in mice [18]. These results could serve as an external validation of the PBPK-PD model due to the accuracy in the prediction of the tumor size reduction, with a negligible difference in the final tumor volumes at the studied concentration of 10 mg/kg.

**Figure 6:**
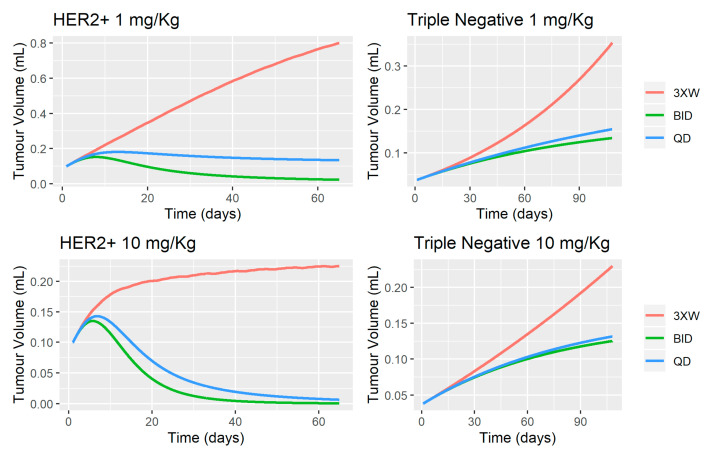
Deterministic simulations to evaluate the impact of intensive dosing strategies on tumor dynamics of HER2+ and Triple Negative cell lines in mice.

The use of quantitative structures for the optimal design of dosage regimens is one of the most relevant applications during the drug development process. In the Simeoni model, *k_1_* and *k_2_* represent the kinetics of cell death and the proportionality factor linking the plasma concentration to the effect (drug potency), respectively [10]. In this sense, deterministic simulations were performed in order to evaluate the influence of intensive dosing strategies on tumor dynamics (Figure 6). The results suggested a significant improvement of tumor reduction when once daily (QD) and/or twice daily (BID) schedules were considered, especially in the HER2+ cell line since tumor eradication is predicted at 1 mg/kg BID and 10 mg/kg BID or QD. The dose-dependent tumor shrinkage observed in the HER2+ cell line is negligible in the Triple Negative cell line, suggesting that maximal pharmacodynamic response is achieved at 1 mg/kg, but tumor stabilization is observed when BID or QD schedules are considered. The accumulation of MBQ-167 in plasma achieved with BID or QD schedule explains the net greater effect observed in tumor dynamics and the significant improvement in terms of tumor eradication (HER2+) or tumor stabilization (Triple Negative). In this sense, the current PBPK-PD model allowed to quantitatively characterize system – (λ_0_, λ_1_, initial tumor volume, and Ψ), which regulate the exponential and linear tumor growth for each cell line, and drug-related (*IC*_50_, *K*_max_, and *k*_1_) parameters, which helped to guide optimal dosing regimens in future preclinical studies. These analyzes can serve as a basis for experimentally evaluating other dosing strategies that allow characterizing tumor dynamics with greater precision, achieving significant reductions in tumor size in both cell lines.

Among the most relevant limitations of the present work, we highlight the lack of an intravenous group, which would have allowed a more precise characterization of the disposition of MBQ-167. Secondly, the impossibility of characterizing the individual PBPK and PBPK-PD profile and, therefore, the inter-individual random components on the parameters of the model since Simcyp V19 Animal Simulator does not allow a population approximation of the data for mice. Furthermore, due to the study design, it is important to note that the typical PK profile was obtained from independent mice since it was not possible to gather the concentration in the tissues in the same animal over time. This necessarily increases the variability in the data and enhances parameter uncertainty. A major drawback of the current analysis was the inability to link concentrations in the tumor tissue as the driving force of the tumor dynamics and the negligible drug effect observed in the Triple Negative cell line when 1 or 10 mg/kg dose regimens were considered, which resulted in an *IC*_50_ = 10^−4^ µM. On the other hand, the current model is limited only to the experimental data available in mice without preclinical information in other animal models. Prospective analyses are encouraged to externally validate predictions of the current PBPK-PD model.

## 5. Conclusions

In summary, we have been able to successfully develop a PBPK-PD model of MBQ-167 in mice that accurately characterizes: (i) the pharmacokinetic properties of MBQ-167 in different mouse tissues, (ii) the dynamics of tumor progression, and (iii) the anti-tumor effect of MBQ-167 in HER2+ and Triple Negative breast tumors following intraperitoneal administration. Moreover, the optimal dosing strategy analysis predicted tumor eradication in HER2+ and tumor stabilization in Triple Negative cell lines when intensive schedules (BID and QD) were selected, despite the higher potency of MBQ-167 in Triple Negative vs. HER2+ cell line. The findings of this study further support the development of MBQ-167 as a therapeutic for breast cancer treatment.

## Figures and Tables

**Table 1:** Parameters of the PBPK model for MBQ-167.

Model Section	Parameter (Units)	Value	Source/Reference/Comments
	Molecular Weight (g/mol)	338.414	
	log*P*_o:w_	4.944	
	Compound Type	Monoprotic Base	
	p*K*_a_	0.27	
	*B/P*	1.8	Optimized
	*f_u_* plasma	0.02	Estimated
**Distribution**	Full PBPK		
	*V_ss_* (L/kg)	20.21	Simcyp predicted (Method 2)
	*Kp_t_* Heart	1	Optimized
	*Kp_t_* Kidney	13.94	Optimized
	*Kp_t_* Liver	14.66	Optimized
	*Kp_t_* Lung	1.9	Optimized
	*Kp_t_* Spleen	2.1	Optimized
	*Kp_t_* Scalar	0.29	Optimized
**Tumor**	Permeability Limited Model		
	*PS* (mL/min/mL tumor volume)	1.2	Optimized
	Efflux transporter (mL/min mL tumor)	7	Estimated
**Elimination**			
	Hep intrinsic *CL* (mL/min/10^6^ cells)	79	In vitro determined
	*f_u_inc_*	0.07	Optimized
	Typical Renal *CL* (mL/min)	0.3	Assumed based on maximum glomerular filtration rate for mice [26].
	Tumor *CL* (mL/min/mL tumor volume)	2.2	Optimized
**Administration Route**	Other site		
	Dose (mg)	0.2	
	Condition	Fed	
	Input site	Venous Blood	Optimized
	Input model	First order	Optimized
	Lag time (h)	0.17	Optimized
	*f_a_*	1	
	*k_a_* (1/h)	3	Optimized

*Vss*: volume of distribution at steady-state. *Kp_t_*: tissue-to-plasma partition coefficient. *CL*: clearance. *PS*: Passive permeability clearance between intra- and extracellular water. Hep: Hepatocytes. *fu_inc*: fraction of unbound drug into the in vitro incubation. *ka*: absorption rate constant. *fa*: fraction of dose absorbed.

**Table 2:** Parameters of the tumor growth (unperturbed and perturbed) model.

Parameter\Cell Line	HER2+	Triple Negative
**System Related Parameters**		
Tumor growth model	Simeoni	Simeoni
Initial tumor volume (mL)	0.1 ^a^	0.0384 ^b^
λ_0_ (day^−1^)	0.2 ^c^	0.0393 ^b^
λ_1_ (g/day)	0.12 ^c^	0.5457 ^b^
Ψ	0.7 ^c^	0.9985 ^b^
Number of transit compartments	3	4
**Drug Related Parameters**		
Response Model	*E*_max_	*E*_max_
Drug input	Total plasma concentration	Total plasma concentration
*k*_1_ (day^−1^)	0.39 ^c^	0.0007 ^b^
*IC*_50_ (μM)	0.0187 ^b^	0.0001 ^b^
*K*_max_ (day^−1^)	0.3683 ^b^	0.0533 ^c^
*H*	0.5 ^c^	0.5 ^c^

^a^: assumed. ^b^: estimated. ^c^: optimized to best fit the observed data. λ_0_: exponential growth rate. λ_1_: linear growth rate. *k*_1_: transit rate of cell damage. Ψ : shape factor. *K*_max_: maximum inhibition. *IC*_50_: concentration associated with *K*_max_/2. *H*: Hill coefficient.

**Table 3:** *AUC*_0-t_ and *C*_max_ values of MBQ-167 with the corresponding fold error after IP single dose administration of 10 mg/kg of MBQ-167 in mice.

Tissue	*AUC*_obs_	*AUC*_pred_	*AUC*_pred_/*AUC*_obs_	*C*_max,obs_	*C*_max,pred_	*C*_max,pred_/*C*_max,obs_
Plasma	1417.2	1549.1	1.09	839.9	833.31	0.99
Lung	1887.6	1838.2	0.97	1540.9	1583.3	1.03
Liver	8388.5	11,444.3	1.36	4793.7	4865.6	1.01
Spleen	3983.9	3243.5	0.81	1693.4	1718.4	1.01
Kidneys	34279	21,527.9	0.63	11,160.4	11,231.7	1.00
Heart	949.2	965.4	1.02	840.8	831.2	0.99
Tumor	10,286.8	11,492.8	1.12	1243.6	997.0	0.8

*AUC*_0-t_ (ng/mL·h); *C*_max_ (ng/mL). Observed (*AUC*_obs_ and *C*_max,obs_) and predicted (*AUC*_pred_ and *C*_max,pred_) PK parameters are calculated within the same time interval.

## Supplementary Materials

The following are available online at https://www.mdpi.com/1999-4923/12/10/975/s1, Figure S1. MBQ-167 PBPK-PD modelling strategy.

## Abbreviations

95%CI	95% confidence intervals
B/P	blood-to-plasma ratio
CL_R_	renal clearance
f_u_	unbound fraction
*IC*_50_	concentration related to half of the maximal inhibitory concentration
IP	intraperitoneal
LSA	local sensitivity analysis
PBPK	Physiologically based pharmacokinetic
PBPK-PD	Physiologically based pharmacokinetic/pharmacodynamic
TGI	tumor growth inhibition
Vss	volume of distribution at steady state
