# Supplementary material for: Physiologically-Based Pharmacokinetic/Pharmacodynamic Model of MBQ-167 to Predict Tumor Growth Inhibition in Mice
Source: Pharmaceutics. 2020 Oct 15;12(10):975. doi: 10.3390/pharmaceutics12100975 (PMC7602742; doi:10.3390/pharmaceutics12100975)
Supplement: Supplementary file 1 [file pharmaceutics-12-00975-s001.zip › pharmaceutics-955694-supplementary.docx]

Supplementary Material: Physiologically-based Pharmacokinetic/ Pharmacodynamic Model of MBQ-167 to Predict Tumor Growth Inhibition in Mice

Javier Reig-López, María del Mar Maldonado, Matilde Merino-Sanjuan, Ailed M. Cruz-Collazo, Jean F. Ruiz-Calderón, Victor Mangas-Sanjuán, Suranganie Dharmawardhane and Jorge Duconge


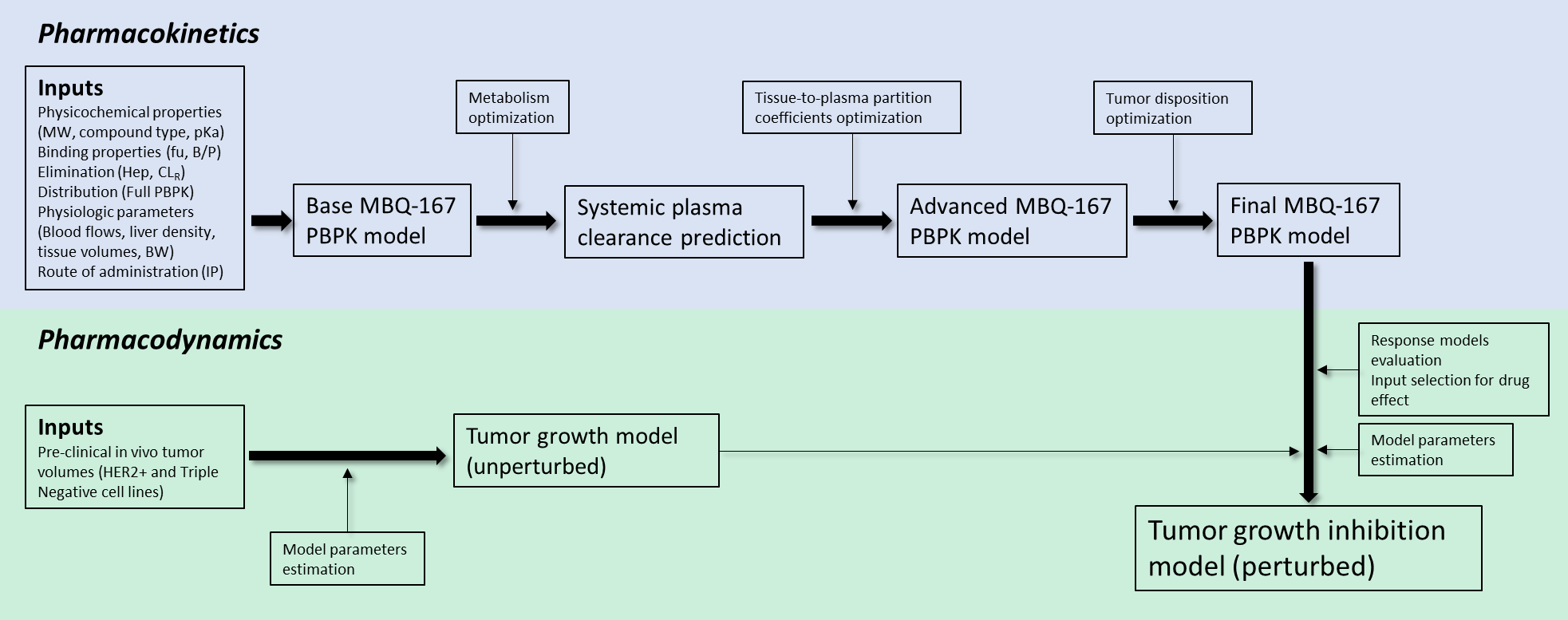
.

**Figure S1.** MBQ-167 PBPK-PD modelling strategy.
